# Association between Lipid Profiles and the Incidence of Hepatocellular Carcinoma: A Nationwide Population-Based Study

**DOI:** 10.3390/cancers13071599

**Published:** 2021-03-30

**Authors:** Yuri Cho, Eun Ju Cho, Jeong-Ju Yoo, Young Chang, Goh Eun Chung, Su-Min Jeong, Sang-Hyun Park, Kyungdo Han, Dong Wook Shin, Su Jong Yu

**Affiliations:** 1 Center for Liver and Pancreatobiliary Cancer, National Cancer Center, Goyang 10408, Korea; yuricho@ncc.re.kr; 2 Department of Internal Medicine, CHA Gangnam Medical Center, CHA University School of Medicine, Seoul 06135, Korea; 3 Department of Internal Medicine and Liver Research Institute, Seoul National University College of Medicine, 101 Daehak-ro, Jongno-gu, Seoul 03080, Korea; creatio3@snu.ac.kr; 4 Department of Gastroenterology and Hepatology, Soonchunhyang University Bucheon Hospital, Bucheon 14584, Korea; puby17@naver.com; 5 Department of Gastroenterology and Hepatology, Soonchunhyang University Seoul Hospital, Seoul 04401, Korea; chyoung@schmc.ac.kr; 6 Department of Internal Medicine, Healthcare Research Institute, Gangnam Healthcare Center, Seoul National University Hospital, Seoul 06236, Korea; gohwom@snu.ac.kr; 7 Department of Family Medicine, Seoul Metropolitan Government-Seoul National University Boramae Medical Center, Seoul 07061, Korea; dpsme@snu.ac.kr; 8 Department of Nutrition, Harvard T.H. Chan School of Public Health, Boston, MA 02115, USA; 9 Department of Biostatistics, College of Medicine, The Soongsil University, Seoul 06978, Korea; ujk8774@catholic.ac.kr (S.-H.P.); hkd@ssu.ac.kr (K.H.); 10 Department of Family Medicine, Samsung Medical Center Supportive Care Center, Samsung Comprehensive Cancer Center, Seoul 06351, Korea; 11 Department of Digital Health, SAIHST, Sungkyunkwan University; 81 Irwon-Ro Gangnam-gu, Seoul 06351, Korea

**Keywords:** cholesterol, low-density lipoprotein, hepatocellular carcinoma, lipid metabolism

## Abstract

**Simple Summary:**

Cholesterol plays an important role in cell structure and cell proliferation. Altered lipid metabolism have been implicated in the development of hepatocellular carcinoma (HCC). This study investigated the relationships between lipid profiles and HCC development using large-scale, nationally representative data from the Korean National Health Insurance Service. During a median of 7.3 years follow-up, 26,891 incident HCCs were identified. The incidence of HCC gradually decreased according to the increase of total-cholesterol and LDL-cholesterol. This inverse association was consistent across subgroups stratified by the presence of liver cirrhosis or viral hepatitis. This large nationwide population-based study suggests that low lipid profile is an independent risk factor and preclinical marker for HCC.

**Abstract:**

Background and Aims: Altered lipid metabolism has been implicated in the development of hepatocellular carcinoma (HCC). This study investigated the relationships between lipid profiles and HCC development. Methods: Data were obtained from the Korean National Health Insurance Service from 2009 to 2017. Cox regression analysis was used to examine the hazard ratios of HCC in 8,528,790 individuals who had undergone health check-ups in 2009. Results: During a median of 7.3 years follow-up, 26,891 incidents of HCCs were identified. The incidence of HCC (per 100,000 person-years) gradually decreased according to the increase in total-cholesterol and LDL-cholesterol; the incidence of HCC was 69.2, 44.0, 33.9, and 25.8 in quartile-1 (Q1), Q2, Q3, and Q4 population of total-cholesterol, and 63.6, 44.5, 37.2, and 28.3 in Q1, Q2, Q3, and Q4 population of LDL-cholesterol, respectively. Compared to Q1 of total-cholesterol, subjects in higher total-cholesterol levels were associated with a lower incidence of HCC (multiple covariates-adjusted hazard ratio (aHR): Q2 0.61; Q3 0.46; Q4 0.36). These associations were consistently observed in stratified subgroup analysis by the presence of liver cirrhosis or viral hepatitis. Conclusions: Low serum lipid levels were significantly associated with the increased risk of developing HCC. A low lipid profile might be an independent risk factor and preclinical marker for HCC.

## 1. Introduction

Cholesterol is a major component of the cell membrane and plays an important role in cell structure and cell proliferation [1]. Cholesterol biosynthesis and metabolism have been implicated in the process of carcinogenesis [2]. A Mendelian meta-analysis reported that a 1 mg/dL reduction in high-density lipoprotein cholesterol (HDL-C) level was associated with a 14% increased risk of overall cancer [3]. Benn et al. reported a Mendelian randomization study suggesting that low-density lipoprotein cholesterol (LDL-C) level was significantly associated with an increased risk of cancer, whereas genetically low LDL-C level did not show an association [4].

In many reports of hepatocellular carcinoma (HCC) patients, serum total cholesterol (TC), triglycerides (TG), free fatty acids (FFA), HDL, LDL-C, and lipoprotein levels were slightly to significantly decreased at baseline [5,6,7]. Liver is a key organ in lipid metabolism and is involved in the production of apolipoproteins, endogenous lipids, and lipoproteins [8]. Serum lipid profiles can be altered in patients with chronic liver disease. Some studies have suggested that lipid disorders in patients with liver disease reflect the status of hepatic cellular impairment [9].

Although prior studies have reported epidemiological correlations between cholesterol levels and HCC, it is hard to generalize the causality for these findings. The numbers of subjects in previous studies were small [5]. Subjects were often retrospectively analyzed after the diagnosis of HCC [10], and the studies did not adjust for multiple confounding factors [11]. We hypothesize that low serum cholesterol levels are associated with the increased risk of HCC in the general population. We analyzed large-scale data from the Korean National Health Insurance Service (NHIS) to evaluate this association.

## 2. Methods

### 2.1. Data Source

Approximately 97% of South Koreans are under a national health insurance system which is mandatory. The NHIS reimburses pharmacies and medical providers based on claims. It also performs biennial health screenings for all employees. The examinations consist of anthropometric measurements, laboratory tests, and questionnaires on lifestyle behaviors. This database has been analyzed in many epidemiologic studies [12,13].

### 2.2. Ethics Statement

This study was approved by the Institutional Review Board of Seoul National University Hospital (IRB no. E-1912-024-1085). Anonymized and de-identified information was used for analyses.

### 2.3. Study Population

Among 10,490,491 individuals (age ≥ 20 years) who underwent health examinations with the Korean National Health Insurance Service in 2009, subjects with missing data (*n* = 676,995), those with pre-existing cancer (*n* = 160,347), and those taking statin medication (*n* = 1,124,359) were excluded. We excluded all subjects taking statin at baseline to minimize the confounding effect. Finally, 8,528,790 subjects were included in the analysis. The last follow-up date was December 2017.

### 2.4. Definition of HCC

HCCs were confirmed using the following diagnosis from the International Classification of Diseases 10th revision (ICD-10): hepatocellular carcinoma (C22.0). Additionally, we identified subjects of HCC by the cancer registration program. Since 2005, the Korean government has provided reductions in payment for registered cancer patients who are diagnosed and registered after complete evaluation.

### 2.5. Measurement of Cholesterol Levels

On the day of the health screening, serum samples were collected after 8 h of fasting. The concentrations of cholesterol were measured enzymatically. If the TG level was <400 mg/dL, the LDL-C level was calculated using the Friedewald Equation [14]. The LDL-C level was measured with direct assay when the TG level was >400 mg/dL. Study subjects were classified by quartile of HDL-C level (quartile 1 (Q1) as the lowest, Q2, Q3, and Q4 as the highest, respectively).

### 2.6. Covariates

Household income was categorized into quartiles depending on health insurance premium. Waist circumference (WC) was measured in a horizontal plane around the abdomen at the level just above the uppermost lateral border of the iliac crest, just below the lowest rib, and midway between both sites. Body mass index (BMI) was calculated as weight in kilograms divided by height in meters squared.

Physical activity, smoking, and alcohol consumption were evaluated by self-reporting questionnaires. “Current smoker” was defined as smoking >5 packs per day currently, or a total of >100 cigarettes, during lifetime. Those who had smoked >5 packs of cigarettes a day but had stopped were defined as “ex-smoker”. A subject who drinks more than 30 g of alcohol a day was defined as “heavy drinker”. “Moderate drinker” was defined as those drink <30 g of alcohol per day. Exercising strenuously ≥1 time per week for at least 20 minutes for one session were defined as “regular physical activity”.

The diagnosis of diabetes mellitus (DM), hypertension, and dyslipidemia was assessed using comprehensive information on health examination data, clinical diagnosis (ICD-10 codes), and pharmacy. The following ICD-10 codes were used: DM (E11–E14), hypertension (I10–I13 and I15), and dyslipidemia (E78). Chronic kidney disease (CKD) was defined as an estimated glomerular filtration rate <60 mL/min per 1.73 m^2^ of body surface area.

Subgroup analyses were performed according to the presence of liver cirrhosis (LC) or viral hepatitis. LC was identified using the following diagnoses from the ICD-10: K703, K746. Viral hepatitis was identified using the following diagnoses from the ICD-10: B15–B19.

### 2.7. Statistical Analysis

Baseline characteristics according to the development of HCC were compared using independent *t*-tests for continuous variables and the chi-square test for categorical variables. The incidence rates of HCC were calculated as the incident cases divided by 100,000 person-years using the Kaplan–Meier method. Cox-proportional hazard regression was performed to estimate the risk of HCC for each quartile of TC, TG, LDL-C, and HDL-C using the lowest quartile as the reference group. Multivariate analyses were adjusted for age, sex, alcohol consumption, smoking history, physical activity, income, BMI, hypertension, DM, and fenofibrate medication.

Sensitivity analyses were performed to deal with possible reverse causality by excluding subjects who expired or were diagnosed as HCC within two years of follow-up. We also conducted a sensitivity analysis after excluding patients who had started statin during the follow-up period to exclude the effects of statin therapy. The potential effect modification by covariates, including the presence of LC or viral hepatitis, was evaluated using stratified analysis. Interaction testing was evaluated using a likelihood ratio test. Statistical analyses were performed using SAS version 9.4 (SAS Institute Inc., Cary, NC, USA). Statistical significance was defined as *p*-value < 0.05.

## 3. Results

### 3.1. Baseline Characteristics

A total of 8,528,790 subjects were enrolled in the study. Among the total subject group, 26,891 individuals (0.32%) developed HCC during a median follow-up of 7.3 years. Baseline characteristics are summarized in Table 1. Subjects in the HCC group were significantly older, with a higher proportion of male and a lower proportion of urban residence than those in the non-HCC group. The HCC group was also significantly associated with higher BMI, wider WC, higher frequency of heavy alcohol drinking, and higher frequency of smoking compared with the non-HCC group. The HCC group also showed a higher prevalence of comorbidities including DM, hypertension, LC, viral hepatitis, and CKD, compared with the non-HCC group. The prevalence of dyslipidemia was lower in the HCC group than in the non-HCC group. In terms of baseline laboratory results, the HCC group had significantly higher serum fasting glucose and lower serum TC, TG, LDL-C, and HDL-C levels compared with the non-HCC group.

### 3.2. Incidence of HCC According to Lipid Profiles

**Figure 1:**
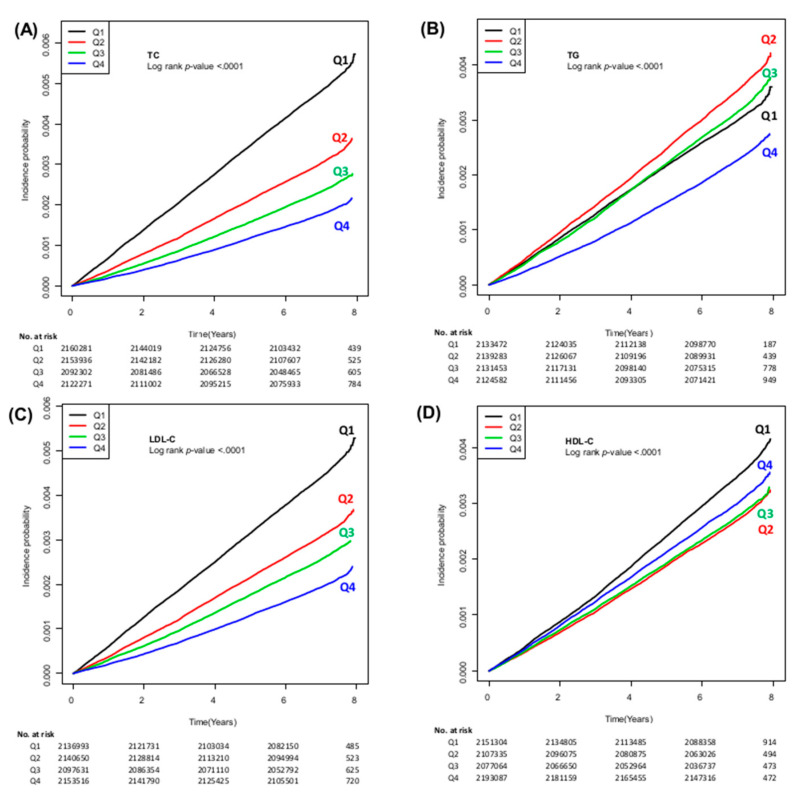
Cumulative incidence of HCC according to the quartiles of TC (**A**), TG (**B**), LDL-C (**C**), and HDL-C (**D**). HCC, hepatocellular carcinoma; TC, total cholesterol; TG, triglycerides; LDL-C, low-density lipoprotein cholesterol; HDL-C, high-density lipoprotein cholesterol.

The incidence of HCC (per 100,000 person-years) gradually decreased according to the increase in TC, TG, LDL-C and HDL-C; the incidence of HCC was 69.2, 44.0, 33.9, and 25.8 in the subjects with Q1, Q2, Q3, and Q4 of TC, respectively. The incidence of HCC was 63.6, 44.5, 37.2, and 28.3 in the population with Q1, Q2, Q3 and Q4 of LDL-C, respectively. The incidence of HCC was 43.2, 51.2, 45.7, and 33.3 in the subjects with Q1, Q2, Q3 and Q4 of TG, respectively. The incidence of HCC was 50.5, 39.2, 40.0, and 43.5 in the subjects with Q1, Q2, Q3 and Q4 of HDL-C, respectively. The cumulative incidences of HCC stratified by each lipid profile are illustrated in Figure 1.

Moreover, the adjusted hazard ratio (aHR) of HCC incidence for Q2, Q3, and Q4 of TC showed a significant and gradual decrease compared with Q1 of TC (Table 2; aHR 0.61, 95% confidence interval (CI) 0.59–0.63; aHR 0.46, 95% CI 0.44–0.47; aHR 0.36, 95% CI 0.34–0.37, respectively). The aHR of HCC also decreased in higher TG with aHRs (95% CI) for HCC in Q2, Q3, and Q4 compared with Q1 of 0.95 (0.91–0.99), 0.77 (0.72–0.82), and 0.59 (0.54–0.65), respectively. The aHR of HCC incidence for Q2, Q3, and Q4 of LDL-C also decreased according to the increase in quartiles of LDL-C compared with Q1 (aHR 0.62, 95% CI 0.60–0.64; aHR 0.48, 95% CI 0.46–0.50; aHR 0.35, 95% CI 0.34–0.36, respectively). Similarly, the aHR of HCC decreased progressively according to the increased quartiles of HDL-C, with aHRs (95% CI) for HCC in Q2, Q3, and Q4 compared with Q1 of 0.84 (0.81–0.86), 0.83 (0.80–0.86), 0.87 (0.84–0.90), respectively (Table 2).

### 3.3. Stratified Analyses by the Presence of LC, Viral Hepatitis or Gender

**Figure 2:**
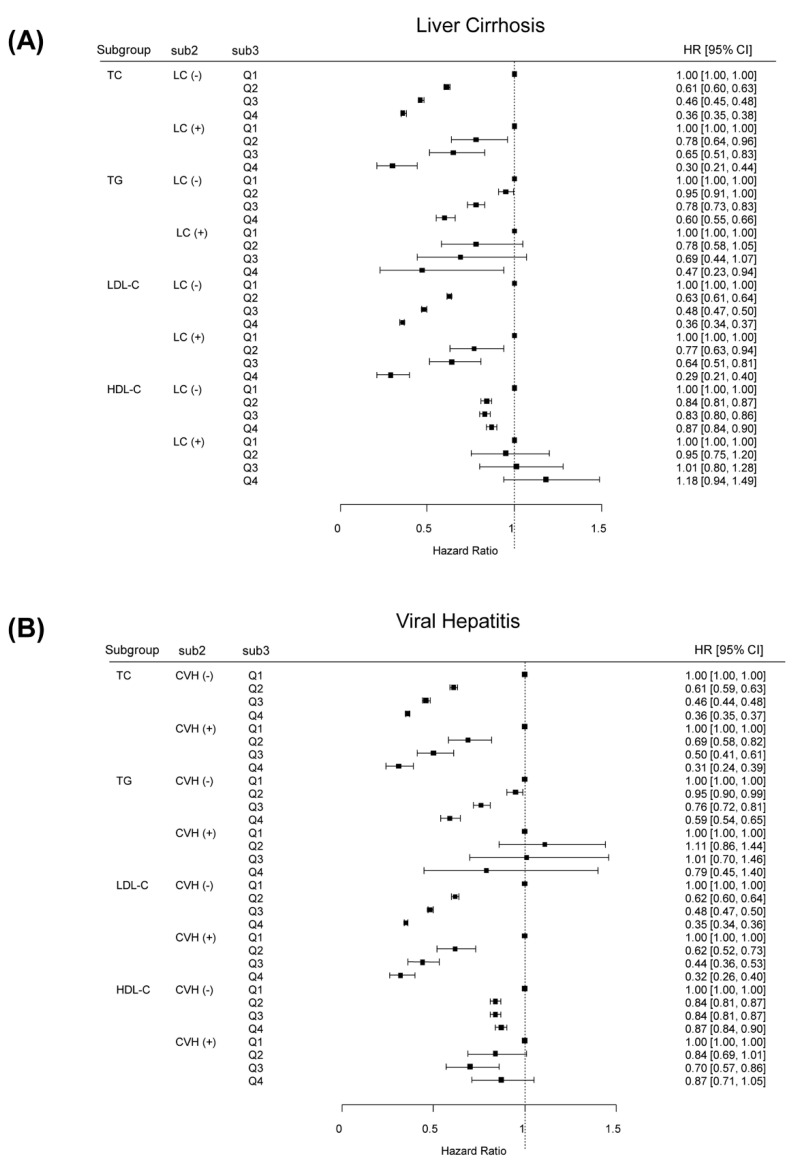
Adjusted HRs of HCC incidence according to lipid profile classification stratified by the presence of LC (**A**) or VH (**B**). HR, hazard ratio; HCC, hepatocellular carcinoma; LC, liver cirrhosis; VH, viral hepatitis.

The association between serum cholesterol levels and the incidence of HCC was investigated in subgroups of the study population stratified by the presence of LC or viral hepatitis (Figure 2). Subgroup analysis stratified by the presence of LC demonstrated similar results. Among patients without LC, the lowest risk of HCC in the highest quartile of serum TC, TG, LDL-C, and HDL-C levels were observed again. Higher serum TC and LDL-C levels were associated with a lower incidence of HCC for patients with LC. We also found similar results in stratified analyses according to the presence of viral hepatitis. Stratified analyses according to gender also revealed similar results among both male and female cohorts (Table S3).

### 3.4. Sensitivity Analysis

In a sensitivity analysis with a two-year lag time, the lowest risk of HCC in the highest quartile of serum lipid levels was observed again (Table S1). The lowest risk of HCC in the highest quartile of serum TC, TG, LDL-C, and HDL-C levels were also found, even after excluding patients who had started statin therapy during the follow-up period (Table S2).

### 3.5. Incidence of HCC According to Lipid Profiles among Subjects Taking Statin

We additionally analyzed 1,124,359 subjects who were taking statin medication at baseline. As shown in Table S4, low serum lipid levels were also associated with an increased incidence of HCC, even among those with statin medication.

## 4. Discussion

In this large, nationwide, population-based study, low serum lipid levels were associated with an increased incidence of HCC. Our data suggest that metabolic dysregulation is one of the risk factors for developing HCC. This inverse association was consistent in stratified analyses by the presence of LC or viral hepatitis. The result of our study might benefit in discriminating subjects who should be under the surveillance for early diagnosis of HCC. The strengths of our study involve a large size of a representative healthy general population using a database from Korean NHIS, leading to sufficient statistical power. We also extensively adjusted multiple potential confounding factors. Moreover, this inverse association was not attenuated in a sensitivity analysis that excluded patients who had started statin therapy during the follow-up period, which means that this finding is independent from the use of statin.

Such an inverse correlation has been suggested in studies of systemic inflammatory diseases, such as rheumatoid arthritis and inflammatory bowel disease [13,15,16]. Although the exact mechanisms are unclear, we suggest several plausible mechanisms. First, dysregulation of cholesterol metabolism itself might act as a part of hepatocarcinogenesis. Cholesterol is involved in numerous biochemical pathways that are potentially relevant in HCC development, including several cytokine and signaling pathways [2]. Interleukin-6, tumor necrosis factor-α, and interleukin-1 inhibit TG synthesis [17]. These proinflammatory cytokines could act as carcinogens or co-factors for hypocholesterolemia and hepatocarcinogenesis [18]. A recent study reported that high cholesterol levels significantly reduced the development and progression of HCC in mice [19]. Cholesterol accumulation reinforced the antitumor property of natural killer cells, a finding which strengthens a new role of cholesterol as an immune-regulator.

Second, low lipid profiles might reflect impaired hepatic function. Approximately 80% of endogenous cholesterol are synthesized in the hepatocellular microsomes [20]. Cholesterol metabolism is impaired in patients with chronic liver disease, leading to a decrease in cholesterol levels [21]. The decrease in LDL-C levels is significantly correlated with the severity of chronic liver disease [6,22,23]. Hence, chronic hepatitis B (CHB) is the most common etiology of HCC in Asia; viral hepatitis could also explain this inverse association [24]. Undiagnosed chronic liver disease might also inhibit cholesterol metabolism [25], possibly exaggerating the negative association with HCC incidence. However, we found similar outcomes even in subjects without LC or viral hepatitis, suggesting that chronic liver disease itself may partially explain this association.

Third, preclinical HCC might itself reduce cholesterol, perhaps by increased receptor activity for LDL-C in HCC cells [26,27]. Low lipid profiles could be a manifestation of malignancy during the latent period. The consumption of cholesterol is doubled in HCC tissues compared with non-tumor tissues [5,28]. Tumor cells induce liposynthesis and accumulation of intracellular cholesteryl esters [29]. The scavenger receptor class B type, an HDL-C receptor, enhances the uptake of cholesteryl esters, leading to a reduction in serum HDL-C levels [30].

Our study has several limitations. First, our study cannot establish a causal relationship, as with all epidemiological studies. However, the sensitivity and stratified analyses suggest that reverse causality is not very likely. We performed sensitivity analyses with a two-year lag and found similar results. A second limitation is that our study analyzed the data among South Koreans, whose incidence of CHB is relatively high. Therefore, the result is not generalizable to other ethnic groups. Regardless, stratified analyses also found similar outcomes among subjects without viral hepatitis. Third, unmeasured factors including diet might still exist as confounders, considering that our data were originally collected for routine clinical purposes.

Although whether low cholesterol levels are biomarkers for HCC remains uncertain, our results warrant further research to determine any causal relationship. Investigating various biochemical pathways that are both involved in cholesterol metabolism and hepatocarcinogenesis may translate into a reasonable explanation for this association. Future investigations should explore whether low serum cholesterol levels can serve as clinical predictors of HCC incidence and outcome. Additionally, future studies evaluating intrahepatic lipid deposits in human liver that contains early HCC might establish the causality.

## 5. Conclusions

In conclusion, this nationwide population-based study demonstrated that low serum TC, TG, LDL-C, and HDL-C levels are associated with increased risk of HCC. A low lipid profile might be a preclinical marker and independent risk factor for HCC. Further prospective well-designed studies are required to establish the biological mechanism by which lipid metabolism plays a role in the development of HCC.

## Figures and Tables

**Table 1:** Baseline characteristics of the study population.

	HCC		
	No	Yes	*p*-Value
*n*	8,501,899	26,891	
Age, years	45.4 ± 13.8	57.8 ± 11.2	<0.0001
Male (%)	4,762,306 (56.1)	21,023 (78.2)	<0.0001
Lowest income quintile (%)	2,255,880 (26.5)	7378 (27.4)	0.0008
Urban residence (%)	3,891,641 (45.8)	12,127 (45.1)	0.03
Smoking status			<0.0001
Never-smoker (%)	4,976,898 (58.5)	11,861 (44.1)	
Ex-smoker (%)	1,183,381 (13.9)	5604 (20.8)	
Current smoker (%)	2,341,620 (27.5)	9426 (35.1)	
Alcohol consumption			<0.0001
Complete or near abstinence (%)	4,191,220 (49.3)	13,054 (48.5)	
Moderate consumption (%)	3,615,635 (42.5)	10,131 (37.7)	
Heavy consumption (%)	695,044 (8.2)	3706 (13.8)	
Regular physical activity (%)	1,496,016 (17.6)	5516 (20.5)	<0.0001
BMI, kg/m^2^	23.5 ± 3.2	24.1 ± 3.1	<0.0001
WC, cm	79.7 ± 9.0	83.8 ± 8.4	<0.0001
SBP, mmHg	121.6 ± 14.7	127.0 ± 15.7	<0.0001
DBP, mmHg	76.0 ± 9.9	78.3 ± 10.2	<0.0001
Comorbidities			
Hypertension (%)	1,775,635 (20.9)	10,815 (40.2)	<0.0001
DM (%)	522,711 (6.2)	5726 (21.3)	<0.0001
Dyslipidemia (%)	808,069 (9.5)	1568 (5.8)	<0.0001
Liver cirrhosis (%)	5838 (0.1)	561 (2.1)	<0.0001
Viral hepatitis (%)	85,562 (1.0)	806 (3)	<0.0001
Chronic kidney disease (%)	427,518 (5.0)	1933 (7.2)	<0.0001
Fenofibrate medication (%)	31,486 (0.4)	141 (0.5)	<0.0001
Laboratory results			
Fasting glucose (mg/dL)	95.5 ± 20.6	106.3 ± 33.5	<0.0001
Total cholesterol (mg/dL)	193.0 ± 34.0	179.4 ± 34.3	<0.0001
TG (mg/dL)	129.2 ± 88.2	119.9 ± 78.1	<0.0001
LDL-C (mg/dL)	111.9 ± 31.5	102.0 ± 31.4	<0.0001
HDL-C (mg/dL)	55.1 ± 13.9	53.1 ± 14.6	<0.0001

HCC, hepatocellular carcinoma; BMI, body mass index; WC, waist circumference; SBP, systolic blood pressure; DBP, diastolic blood pressure; DM, diabetes mellitus; TC, total cholesterol; HDL-C, high-density lipoprotein cholesterol; LDL-C, low-density lipoprotein cholesterol; TG, triglycerides. Values are presented as mean ± standard deviation for continuous variables and number (%) for categorical variables.

**Table 2:** Incidence of hepatocellular carcinoma (HCC) according to lipid profile classification.

	HCC Cases (*n*)	Incidence of HCC (100,000 Person-(100,000 Person-Years)	Crude HR (95% CI)	Adjusted HR ^a^ (95% CI)	*p*-Value
TC (mg/dL)					
Q1 (0–169)	10,839	69.22	1 (reference)	1 (reference)	
Q2 (170–191)	6891	43.98	0.64 (0.62–0.66)	0.61 (0.59–0.63)	<0.0001
Q3 (200–214)	5170	33.94	0.49 (0.47–0.51)	0.46 (0.44–0.47)	<0.0001
Q4 (≥215)	3991	25.84	0.37 (0.36–0.39)	0.36 (0.34–0.37)	<0.0001
TG (mg/dL)					
Q1 (0–72)	6711	43.17	1 (reference)	1 (reference)	
Q2 (73–105)	7969	51.24	1.19 (1.15–1.23)	0.95 (0.91–0.99)	<0.0001
Q3 (106–157)	7078	45.72	1.06 (1.02–1.09)	0.77 (0.72–0.82)	<0.0001
Q4 (≥158)	5133	33.26	0.77 (0.74–0.80)	0.59 (0.54–0.65)	<0.0001
LDL-C (mg/dL)					
Q1 (0–90)	9846	63.58	1 (reference)	1 (reference)	
Q2 (91–110)	6923	44.46	0.70 (0.68–0.72)	0.62 (0.60–0.64)	<0.0001
Q3 (111–131)	5678	37.18	0.58 (0.57–0.60)	0.48 (0.46–0.50)	<0.0001
Q4 (≥132)	4444	28.34	0.45 (0.43–0.46)	0.35 (0.34–0.36)	<0.0001
HDL-C (mg/dL)					
Q1 (0–48)	7891	50.51	1 (reference)	1 (reference)	
Q2 (49–57)	6022	39.24	0.78 (0.75–0.80)	0.84 (0.81–0.86)	<0.0001
Q3 (58–66)	6046	40.0	0.79 (0.77–0.82)	0.83 (0.80–0.86)	<0.0001
Q4 (≥67)	6932	43.53	0.86 (0.84–0.89)	0.87 (0.84–0.90)	<0.0001

HCC, hepatocellular carcinoma; TC, total cholesterol; HDL-C, high-density lipoprotein cholesterol; LDL-C, low-density lipoprotein cholesterol; TG, triglycerides; HR, hazard ratio; CI, confidence interval; BMI, body mass index; DM, diabetes mellitus. ^a^ Multivariable analysis including age, sex, alcohol consumption, smoking history, regular physical activity, income, BMI, hypertension, DM, and current fenofibrate medication.

## Data Availability

Publicly available datasets were analyzed in this study. This data can be found here: (https://nhiss.nhis.or.kr/NHIS-2020-1-303, accessed on 21 February 2020).

## Supplementary Materials

The following are available online at https://www.mdpi.com/article/10.3390/cancers13071599/s1, Table S1: Incidence of HCC according to lipid profile classification with a 2-year lag, Table S2: Incidence of HCC according to lipid profile classification excluding subjects who had started statins during follow-up, Table S3: Incidence of HCC according to lipid profile classification according to gender, Table S4: Incidence of HCC according to lipid profile classification among subjects who had been taking statins at baseline.
