# Supplementary material for: Association between Lipid Profiles and the Incidence of Hepatocellular Carcinoma: A Nationwide Population-Based Study
Source: Cancers (Basel). 2021 Mar 30;13(7):1599. doi: 10.3390/cancers13071599 (PMC8037932; doi:10.3390/cancers13071599)
Supplement: Supplementary file 1 [file cancers-13-01599-s001.pdf]

cancers

Article

Association between Lipid Profiles and the Incidence of Hepatocellular Carcinoma: A Nationwide Population-Based Study

Yuri Cho [1,2,†], Eun Ju Cho [3,†], Jeong-Ju Yoo [4], Young Chang [5], Goh Eun Chung [6], Su-Min Jeong [7,8], Sang-Hyun Park [9], Kyungdo Han [9], Dong Wook Shin [10,11,*] and Su Jong Yu [3,*]

Citation: Cho, Y.; Cho, E.J.; Yoo, J.-J.; Chang, Y.; Chung, G.E.; Jeong, S.-M.; Park, S.-H.; Han, K.; Shin, D.W.; Yu, S.J. Association between Lipid Profiles and the Incidence of Hepatocellular Carcinoma: A Nationwide Population-Based Study. *Cancers* **2021**, *13*, 1599. https://doi.org/10.3390/cancers13071599

Academic Editor: Masahito Shimizu

Received: 5 February 2021
Accepted: 23 March 2021
Published: 30 March 2021

Publisher's Note: MDPI stays neutral with regard to jurisdictional claims in published maps and institutional affiliations.

Copyright: © 2021 by the authors. Submitted for possible open access publication under the terms and conditions of the Creative Commons Attribution (CC BY) license (http://creativecommons.org/licenses/by/4.0/).

Table S1. Incidence of HCC according to lipid profile classification with a 2-year lag.

	HCC cases (*n*)	Incidence of HCC (100,000 person-years)	Crude HR (95% CI)	Adjusted HR[a] (95% CI)	*p*-value
TC					
Q1	10,461	67.87	1 (reference)	1 (reference)	
Q2	6,569	43.03	0.63 (0.62–0.65)	0.61 (0.59–0.63)	<0.0001
Q3	4,793	32.88	0.48 (0.47–0.50)	0.46 (0.44–0.48)	<0.0001
Q4	3,511	24.98	0.37 (0.35–0.38)	0.36 (0.35–0.37)	<0.0001
TG					
Q1	6,529	42.73	1 (reference)	1 (reference)	
Q2	7,602	50.49	1.18 (1.14–1.22)	0.94 (0.90–0.98)	<0.0001
Q3	6,620	44.99	1.05 (1.02–1.09)	0.76 (0.71–0.81)	<0.0001
Q4	4,583	32.13	0.75 (0.72–0.78)	0.57 (0.52–0.63)	<0.0001
LDL-C					
Q1	9,467	62.57	1 (reference)	1 (reference)	
Q2	6,596	43.55	0.70 (0.68–0.72)	0.62 (0.6–0.64)	<0.0001
Q3	5,284	36.09	0.58 (0.56–0.60)	0.47 (0.46–0.49)	<0.0001
Q4	3,987	27.70	0.44 (0.43–0.46)	0.35 (0.34–0.36)	<0.0001
HDL-C					
Q1	7,353	49.9	1 (reference)	1 (reference)	
Q2	5,671	38.69	0.78 (0.749–0.803)	0.84 (0.81–0.87)	<0.0001
Q3	5,732	39.42	0.79 (0.764–0.818)	0.83 (0.80–0.86)	<0.0001
Q4	6,578	42.78	0.86 (0.83–0.888)	0.87 (0.84–0.9)	<0.0001

Abbreviations: HCC, hepatocellular carcinoma; TC, total cholesterol; HDL-C, high-density lipoprotein cholesterol; LDL-C, low-density lipoprotein cholesterol; TG, triglycerides; HR, hazard ratio; CI, confidence interval; BMI, body mass index; DM, diabetes mellitus. [a] Multivariable analysis including age, sex, alcohol consumption, smoking history, regular physical activity, income, BMI, hypertension, DM, and current fenofibrate medication.

Table S2. Incidence of HCC according to lipid profile classification excluding patients who had started statins during follow-up.

	HCC cases (*n*)	Incidence of HCC (100,000 person-years)	Crude HR (95% CI)	Adjusted HR[a] (95% CI)	*p*-value
TC					
Q1	9,915	66.34	1 (reference)	1 (reference)	
Q2	5,963	41.45	0.63 (0.61–0.65)	0.61 (0.59–0.63)	<0.0001
Q3	4,101	31.29	0.47 (0.46–0.49)	0.46 (0.44–0.47)	<0.0001
Q4	2,729	24.12	0.37 (0.35–0.38)	0.36 (0.35–0.38)	<0.0001
TG					
Q1	6,186	42.40	1 (reference)	1 (reference)	
Q2	6,949	50.04	1.18 (1.14–1.22)	0.94 (0.90–0.99)	<0.0001
Q3	5,819	44.37	1.05 (1.01–1.09)	0.76 (0.70–0.81)	<0.0001
Q4	3,754	30.86	0.73 (0.70–0.76)	0.56 (0.50–0.62)	<0.0001
LDL-C					
Q1	8,906	61.51	1 (reference)	1 (reference)	
Q2	6,031	42.31	0.69 (0.67–0.71)	0.62 (0.60–0.64)	<0.0001
Q3	4,596	34.67	0.56 (0.55–0.59)	0.47 (0.45–0.49)	<0.0001
Q4	3,175	26.99	0.44 (0.42–0.46)	0.35 (0.34–0.37)	<0.0001
HDL-C					
Q1	6,487	9.78	1 (reference)	1 (reference)	
Q2	5,017	37.94	0.76 (0.73–0.79)	0.83 (0.80–0.86)	<0.0001
Q3	5,211	39.16	0.79 (0.76–0.82)	0.84 (0.81–0.87)	<0.0001
Q4	5,993	42.23	0.85 (0.82–0.88)	0.87 (0.84–0.91)	<0.0001

Abbreviations: HCC, hepatocellular carcinoma; TC, total cholesterol; HDL-C, high-density lipoprotein cholesterol; LDL-C, low-density lipoprotein cholesterol; TG, triglycerides; HR, hazard ratio; CI, confidence interval; BMI, body mass index; DM, diabetes mellitus. [a] Multivariable analysis including age, sex, alcohol consumption, smoking history, regular physical activity, income, BMI, hypertension, DM, and fenofibrate medication.

Table S3. Incidence of HCC according to lipid profile classification according to gender.

Male	HCC cases (*n*)	Incidence of HCC (100,000 person-years)	Crude HR (95% CI)	Adjusted HR[a] (95% CI)
TC				
Q1	8,770	102.58	1 (reference)	1 (reference)
Q2	5,386	61.52	0.6 (0.58–0.62)	0.62 (0.6–0.64)
Q3	3,919	45.4	0.44 (0.43–0.46)	0.47 (0.45–0.48)
Q4	2,948	33.87	0.33 (0.32–0.34)	0.38 (0.36–0.39)
TG				
Q1	4888	83.74	1 (reference)	1 (reference)
Q2	6,055	76.76	0.92 (0.88–0.95)	0.95 (0.9–0.99)
Q3	5,647	59.66	0.71 (0.69–0.74)	0.78 (0.73–0.84)
Q4	4,433	38.72	0.46 (0.44–0.48)	0.62 (0.55–0.69)
LDL-C				
Q1	7992	90.90	1 (reference)	1 (reference)
Q2	5381	62.37	0.69 (0.66–0.71)	0.62 (0.6–0.65)
Q3	4,385	51.08	0.56 (0.54–0.58)	0.49 (0.48–0.51)
Q4	3,265	37.81	0.42 (0.4–0.43)	0.37 (0.35–0.38)
HDL-C				
Q1	6,010	67.10	1 (reference)	1 (reference)
Q2	4508	55.04	0.82 (0.79–0.85)	0.83 (0.8–0.86)
Q3	4820	55.61	0.83 (0.8–0.86)	0.82 (0.79–0.86)
Q4	5685	64.42	0.96 (0.93–1)	0.86 (0.83–0.9)
**Female**	**HCC cases (*n*)**	**Incidence of HCC (100,000 person-years)**	**Crude HR (95% CI)**	**Adjusted HR (95% CI)**
TC				
Q1	2,069	29.10	1 (reference)	1 (reference)
Q2	1,505	21.76	0.75 (0.7–0.8)	0.58 (0.54–0.62)
Q3	1,251	18.95	0.65 (0.61–0.7)	0.42 (0.39–0.45)
Q4	1,043	15.47	0.53 (0.49–0.57)	0.3 (0.27–0.32)
TG				
Q1	1,823	18.78	1 (reference)	1 (reference)
Q2	1,914	24.98	1.33 (1.25–1.42)	0.97 (0.89–1.07)
Q3	1,431	23.79	1.26 (1.18–1.35)	0.74 (0.64–0.85)

Q4	700	17.57	0.93 (0.85–1.02)	0.52 (0.42–0.64)
LDL-C				
Q1	1,854	27.70	1 (reference)	1 (reference)
Q2	1,542	22.21	0.8 (0.75–0.86)	0.6 (0.56–0.65)
Q3	1,293	19.34	0.7 (0.65–0.75)	0.42 (0.39–0.45)
Q4	1,179	16.73	0.6 (0.56–0.65)	0.29 (0.27–0.32)
HDL-C				
Q1	1,881	28.21	1 (reference)	1 (reference)
Q2	1,514	21.16	0.75 (0.7–0.8)	0.85 (0.8–0.91)
Q3	1,226	19.01	0.68 (0.63–0.73)	0.85 (0.79–0.91)
Q4	1,247	17.57	0.63 (0.58–0.67)	0.88 (0.81–0.94)

Abbreviations: HCC, hepatocellular carcinoma; TC, total cholesterol; HDL-C, high-density lipoprotein cholesterol; LDL-C, low-density lipoprotein cholesterol; TG, triglycerides; HR, hazard ratio; CI, confidence interval; BMI, body mass index; DM, diabetes mellitus. [a] Multivariable analysis including age, sex, alcohol consumption, smoking history, regular physical activity, income, BMI, hypertension, DM, and fenofibrate medication.

Table S4. Incidence of HCC according to lipid profile classification among subjects who had been taking statins at baseline (*n* = 1,124,359).

	HCC cases (*n*)	Incidence of HCC (100,000 person-years)	Crude HR (95% CI)	Adjusted HR[a] (95% CI)	*P*-value
TC					
Q1	1,424	71.35	1 (reference)	1 (reference)	
Q2	963	47.71	0.67 (0.62–0.72)	0.88 (0.81–0.96)	0.004
Q3	736	35.26	0.49 (0.45–0.54)	0.78 (0.71–0.86)	<0.001
Q4	486	23.99	0.34 (0.3–0.37)	0.65 (0.59–0.73)	<0.001
TG					
Q1	1,067	53.03	1 (reference)	1 (reference)	
Q2	967	47.47	0.9 (0.82–0.98)	0.89 (0.79–0.99)	0.039
Q3	787	38.57	0.73 (0.66–0.8)	0.72 (0.61–0.84)	<0.001
Q4	788	38.67	0.73 (0.67–0.8)	0.74 (0.58–0.93)	0.011
LDL-C					
Q1	1,330	65.85	1 (reference)	1 (reference)	
Q2	991	49.01	0.74 (0.68–0.81)	0.9 (0.83–0.98)	0.018
Q3	786	38.16	0.58 (0.53–0.63)	0.83 (0.76–0.91)	<0.001
Q4	502	24.77	0.38 (0.34–0.42)	0.66 (0.59–0.73)	<0.001
HDL-C					
Q1	1,092	52.27	1 (reference)	1 (reference)	
Q2	798	40.68	0.78 (0.71–0.85)	0.81 (0.74–0.89)	<0.001
Q3	811	39.41	0.75 (0.69–0.83)	0.79 (0.72–0.86)	<0.001
Q4	908	44.97	0.86 (0.79–0.94)	0.86 (0.78–0.95)	0.002

Abbreviations: HCC, hepatocellular carcinoma; TC, total cholesterol; HDL-C, high-density lipoprotein cholesterol; LDL-C, low-density lipoprotein cholesterol; TG, triglycerides; HR, hazard ratio; CI, confidence interval; BMI, body mass index; DM, diabetes mellitus. [a] Multivariable analysis including age, sex, alcohol consumption, smoking history, regular physical activity, income, BMI, hypertension, DM, and fenofibrate medication.
